# Supplementary material for: Using the Oral Assessment Guide to Predict the Onset of Pneumonia in Residents of Long-Term Care and Welfare Facilities: A One-Year Prospective Cohort Study
Source: Int J Environ Res Public Health. 2022 Oct 22;19(21):13731. doi: 10.3390/ijerph192113731 (PMC9654310; doi:10.3390/ijerph192113731)
Supplement: Supplementary file 1 [file ijerph-19-13731-s001.zip › English-Editing-Certificate-52286.pdf]

We certify that the following article

Using Oral Assessment Guide to Predict Onset of Pneumonia in Residents of Long-Term Care and Welfare Facilities: A 1-Year Prospective Cohort Study

Kanetaka Yamaguchi

has undergone English language editing by MDPI. The text has been checked for correct use of grammar and common technical terms, and edited to a level suitable for reporting research in a scholarly journal.
MDPI uses experienced, native English speaking editors. Full details of the editing service can be found at
► https://www.mdpi.com/authors/english.

MDPI Multidisciplinary Digital Publishing Institute

Basel, Switzerland
October 2022

AUTHORservices
