# Supplementary material for: Using the Oral Assessment Guide to Predict the Onset of Pneumonia in Residents of Long-Term Care and Welfare Facilities: A One-Year Prospective Cohort Study
Source: Int J Environ Res Public Health. 2022 Oct 22;19(21):13731. doi: 10.3390/ijerph192113731 (PMC9654310; doi:10.3390/ijerph192113731)
Supplement: Supplementary file 1 [file ijerph-19-13731-s001.zip › reviceüjTablesS1 ver4.pdf]

Table S1. The number of people in Exclusion criteria by Long-Term Care and Welfare Facilities

Long-Term Care and Welfare Facilities	The number of people in Exclusion criteria	
	Female	Male
1	1	0
2	0	0
3	3	0
4	0	0
5	0	0
6	0	0
7	0	0
8	1	1
9	3	2
