# Supplementary material for: Using the Oral Assessment Guide to Predict the Onset of Pneumonia in Residents of Long-Term Care and Welfare Facilities: A One-Year Prospective Cohort Study
Source: Int J Environ Res Public Health. 2022 Oct 22;19(21):13731. doi: 10.3390/ijerph192113731 (PMC9654310; doi:10.3390/ijerph192113731)
Supplement: Supplementary file 1 [file ijerph-19-13731-s001.zip › reviceüjTablesS2 ver4.pdf]

Table S2. Participant dropout rate by Long-Term Care and Welfare Facilities

Long-Term Care and Welfare Facilities	Dropout rate (%)
1	48.3
2	26.3
3	22.5
4	40
5	20
6	10.3
7	26.3
8	23.3
9	25
