# Supplementary material for: Using the Oral Assessment Guide to Predict the Onset of Pneumonia in Residents of Long-Term Care and Welfare Facilities: A One-Year Prospective Cohort Study
Source: Int J Environ Res Public Health. 2022 Oct 22;19(21):13731. doi: 10.3390/ijerph192113731 (PMC9654310; doi:10.3390/ijerph192113731)
Supplement: Supplementary file 1 [file ijerph-19-13731-s001.zip › reviceüjTablesS3 ver4.pdf]

Table S3.The number of onset of pneumonia by Long-Term Care and Welfare Facilities

Long-Term Care and Welfare Facilities	The number of onset of pneumonia	
	Female	Male
1	0	2
2	0	1
3	0	0
4	0	0
5	0	0
6	2	3
7	0	0
8	0	0
9	0	0
