# Supplementary material for: Using the Oral Assessment Guide to Predict the Onset of Pneumonia in Residents of Long-Term Care and Welfare Facilities: A One-Year Prospective Cohort Study
Source: Int J Environ Res Public Health. 2022 Oct 22;19(21):13731. doi: 10.3390/ijerph192113731 (PMC9654310; doi:10.3390/ijerph192113731)
Supplement: Supplementary file 1 [file ijerph-19-13731-s001.zip › reviceüjTablesS4 ver4.pdf]

Table S4. Assessing normality for Age and Medicine

	P value
Age*	0.001
Medicine*	0.009

Kolmogorov-Smirnov test with Lilliefors correction

* $p < 0.01$
