# Supplementary material for: Using the Oral Assessment Guide to Predict the Onset of Pneumonia in Residents of Long-Term Care and Welfare Facilities: A One-Year Prospective Cohort Study
Source: Int J Environ Res Public Health. 2022 Oct 22;19(21):13731. doi: 10.3390/ijerph192113731 (PMC9654310; doi:10.3390/ijerph192113731)
Supplement: Supplementary file 1 [file ijerph-19-13731-s001.zip › reviceüjTablesS5 ver4.pdf]

Table S5. Model Fitting of Multiple logistic regression analysis

	P value
Model 1	0.843
Model 2	0.565
Model 3	0.964

hosmer-lemeshow test
